# Systemic Lupus Erythematosus Lymphadenopathy Presenting as Kikuchi-Fujimoto Disease in an Adolescent

**DOI:** 10.7759/cureus.35304

**Published:** 2023-02-22

**Authors:** Jessi Harrison, Sukesh Sukumaran, Vini Vijayan

**Affiliations:** 1 Pediatrics, Valley Children’s Healthcare, Madera, USA; 2 Pediatric Rheumatology, Valley Children’s Healthcare, Madera, USA; 3 Pediatric Infectious Diseases, Valley Children's Healthcare, Madera, USA

**Keywords:** lupus lymphadenitis, systemic lupus erythematosus, necrotizing lymphadenitis, kikuchi, kikuchi-fujimoto, lymphadenitis

## Abstract

Systemic lupus erythematosus is a multisystem autoimmune disorder with a highly heterogeneous clinical presentation. The clinical phenotype varies from mild cutaneous and musculoskeletal manifestations to neurological involvement. Lymphadenopathy is a frequent manifestation of SLE, but the association is often not recognized, as lymphadenopathy is not a criterion for diagnosis. An unusual and seldom reported mimicker of lupus lymphadenitis is Kikuchi-Fujimoto Disease. This is a rare self-limiting disease of young adult females that presents with lymphadenopathy, fever, and systemic symptoms. Lupus lymphadenitis and KFD share some common clinical and pathologic features; but distinguishing between those two diseases can be challenging.

We describe a 16-year-old Hispanic female who presented with axillary lymphadenopathy and was initially diagnosed with KFD based on an excisional lymph node biopsy; but later met the criteria for the 2019 European League Against Rheumatism/American College of Rheumatology classification criteria SLE. This case highlights the need for clinicians to be aware that patients with SLE may present with lymphadenopathy and to consider the association between Kikuchi disease and SLE to prevent misdiagnosis and allow for timely treatment to avoid complications.

## Introduction

Systemic lupus erythematosus (SLE) is a multisystem autoimmune disorder with a highly heterogeneous clinical presentation. The clinical phenotype varies from mild cutaneous and musculoskeletal manifestations to neurological and neuropsychiatric involvement [1]. The diagnosis of SLE is based on the 2019 European League Against Rheumatism/American College of Rheumatology classification criteria. This criterion includes a positive antinuclear antibody (ANA) titer and seven clinical criteria (constitutional, hematologic, neuropsychiatric, mucocutaneous, serosal, musculoskeletal, renal) and three immunologicals (antiphospholipid antibodies, complement proteins, SLE-specific antibodies) criteria. Each criterion is weighted, and patients with >10 points are classified. Although lymphadenopathy is a frequent manifestation of SLE, it is not considered a criterion for diagnosis [2]. 

The exact prevalence of lymphadenopathy in children with SLE is unknown. Shapira et al. reported the prevalence of lymphadenopathy in patients with SLE to be 23%-34%, with a female preponderance. Lymphadenopathy may be localized or generalized and often involves the cervical, axillary, and inguinal regions. The involved nodes are typically soft and non-tender, and vary in size from 0.5 to several centimeters. Lymph node involvement has been reported to appear during the onset of the disease or relapses. Patients with lymphadenopathy and SLE have been shown to demonstrate significantly higher disease activity as characterized by more constitutional symptoms, including fatigue, fever, and weight loss. These patients also have a propensity for cutaneous involvement, elevated anti-dsDNA antibody titers, and lower complement levels [2-4].

In this report, our patient initially presented with lymphadenopathy and had not met the criteria for SLE at the time of discharge. Due to the presence of systemic symptoms and laboratory abnormalities, she underwent a lymph node biopsy that demonstrated pathologic features of Kikuchi-Fujimoto’s Disease.

## Case presentation

A previously healthy 16-year-old Hispanic female presented to the emergency department (ED) with fever and left side axillary pain, and swelling for one month. Her mother reported that the child had daily fevers as high as 39°C. Fevers were intermittent and associated with chills and rigors. The child endorsed profuse night sweats, fatigue, and a 5-pound weight loss. There was no history of rhinorrhea, cough, or dental pain. The child denied dysphagia, odynophagia, dysphonia, or otalgia. She was evaluated by her primary care physician and received amoxicillin-clavulanic acid for 10 days for presumed bacterial lymphadenitis. However, the fevers persisted, and the axillary mass continued to increase in size. The child developed a non-pruritic rash on the arms and trunk on the day of the presentation, which prompted the family to bring the child to the ED. Past medical history, surgical history, and family history were non-contributory.

The family denied any history of travel, but the child had exposure to cats, parakeets, and a hamster. She denied any sick contacts, ingestion of unpasteurized dairy products, or tuberculosis exposures. Her immunizations were up to date. 

Physical examination revealed a pale, ill-appearing child with a temperature of 38.9°C, heart rate of 120 beats per minute, respiratory rate of 20 breaths per minute, blood pressure of 116/72 mm of Hg, and oxygen saturation of 99%. Examination revealed a tender lymph node in the left axilla that was approximately 5 cm in size. The lymph node was firm, non-matted, and warm to the touch, with overlying erythema but no fluctuance. She had no other palpable lymphadenopathy on examination. She had a diffuse, erythematous, macular rash that involved her trunk, extremities, and bilateral cheeks, sparing the nasolabial folds. The abdomen was soft but distended without hepatosplenomegaly. The rest of the examination was normal.

Initial laboratory evaluation demonstrated a white blood cell count of 2.5 x 109/L with 74% neutrophils, 14% lymphocytes, and 5% monocytes. Her hemoglobin was 8.8 gm/dL, and her platelet count was 180 x10 9/L. Liver function tests were within normal limits, and lactate dehydrogenase was elevated at 330 U/L. Her C-reactive protein and erythrocyte sedimentation rate were elevated at 18.7 mg/L and 73 mm/h, respectively. A contrast-enhanced computed tomography of the neck, chest, abdomen, and pelvis showed an abnormally enlarged left-sided axillary lymph node and cervical, hilar, and mediastinal lymph nodes. The child was admitted for further evaluation and management.

**Figure 1:**
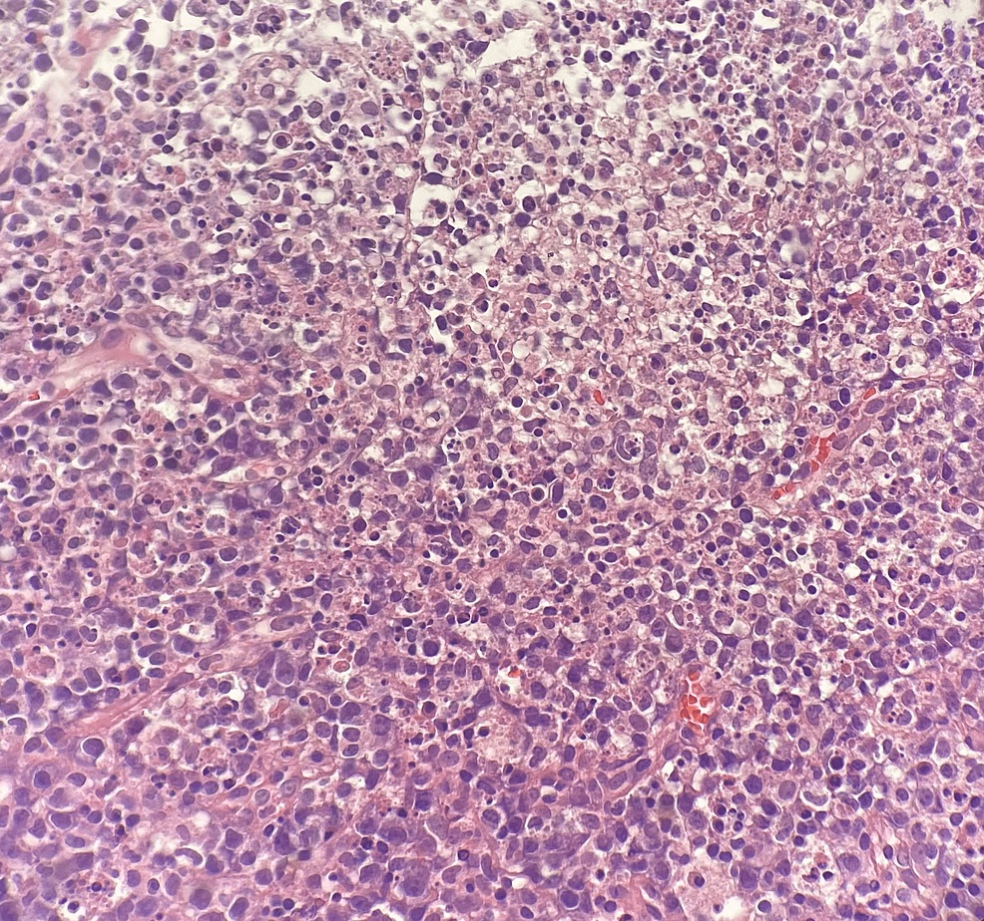
Hematoxylin-eosin-stained section (10X) show axillary lymph node with architecture distorted by pale areas composed of eosinophilic, granular material, karyorrhectic debris and interspersed lymphocytes and histiocytes. No hematoxylin bodies or aggregates of large atypical lymphocytes are identified.

**Figure 2:**
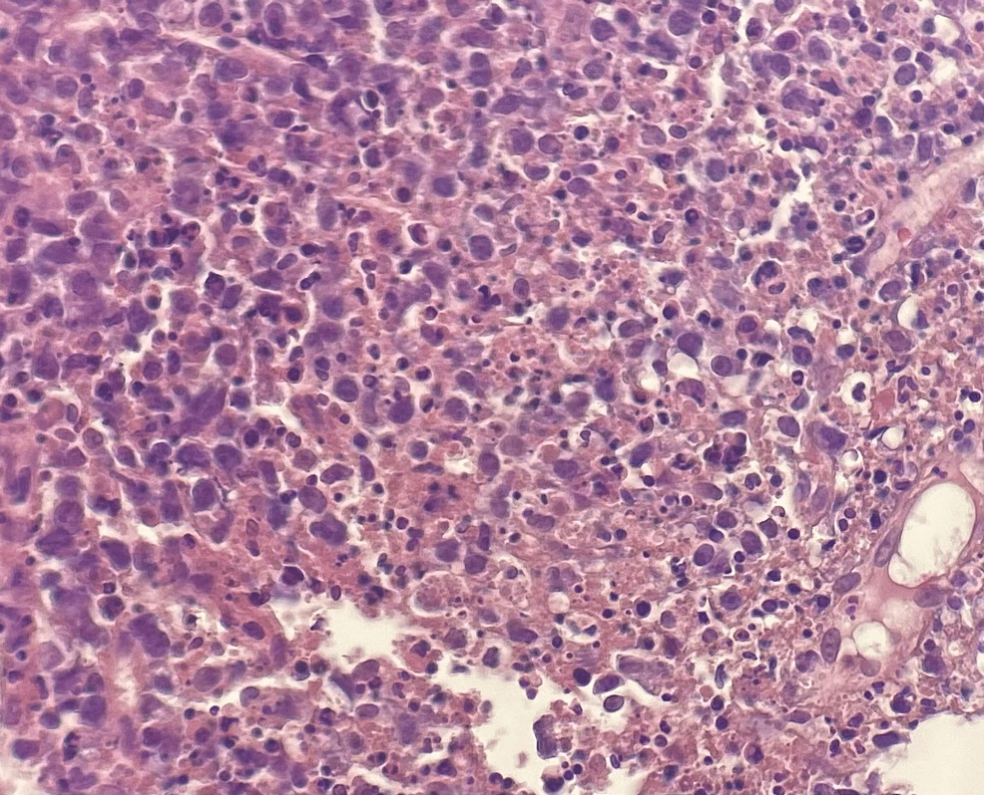
Hematoxylin-eosin-stained section (400x) of the axillary lymph node show lymphocytes with areas of necrosis, abundant karyorrhectic debris, and numerous histiocytes.

Upon admission, the child’s differential diagnosis included malignancy, particularly lymphoma, bacterial lymphadenitis, cat-scratch disease, toxoplasmosis, syphilis, sarcoidosis, and lupus lymphadenitis. Serological testing for human immunodeficiency virus, Epstein-Barr virus, cytomegalovirus, *Bartonella henselae,* toxoplasma, *Brucella *sp., and parvovirus were negative. Due to the high suspicion of lymphoma, an excisional lymph node biopsy was performed for histopathological diagnosis. The lymph node biopsy showed destruction of the nodal architecture and focal necrosis of cortical and paracortical areas with karyorrhexis, crescentic histiocytes, and plasmacytoid monocytes. Immunohistochemical staining demonstrated CD68-positive histocytes and CD8-positive T-cells, characteristic of KFD. No neutrophils, granuloma, or vasculitis were evident. Periodic acid Schiff, Ziehl-Neelsen, and Gram stains were negative for fungus, acid-fast bacilli, and bacteria, respectively. Based on these histopathological findings, the diagnosis of Kikuchi-Fujimoto disease was made. She was treated symptomatically with nonsteroidal anti-inflammatory drugs, clinically improved, and was discharged home (Figures 1, 2). 

At her clinic follow-up visit one week later, the patient reported a resolution of fever and a decrease in the size of her axillary lymph node. However, her facial rash was still visible, and the child reported new onset of swelling and pain in her bilateral ankles, wrists, and knees. Her antinuclear antibody (ANA) was positive with a homogeneous pattern and titer of 1:640. Anti-double-stranded DNA (ds-DNA), anti-Smith, and anti-ribonucleic protein were positive. Anti-Ro antibodies and anti-La, lupus anticoagulant, and anticardiolipin antibodies were negative. Her complements were low.

The child was diagnosed with SLE based on the 2019 European League Against Rheumatism/American College of Rheumatology classification criteria [5]. She was initiated on an immunosuppression regimen which consisted of hydroxychloroquine, prednisone, and mycophenolate mofetil. The patient improved clinically with nonsteroidal anti-inflammatory drugs, and the lymph nodes regressed over two months. She was diagnosed with Kikuchi-Fujimoto's disease and systemic lupus erythematosus. She remains in clinical remission six months after her initial presentation.

## Discussion

The Kikuchi-Fujimoto disease is also known as histiocytic necrotizing lymphadenitis and was initially described in Japan by Kikuchi and Fujimoto in 1972 [6]. This is a benign and self-limited lymphadenopathy that predominantly affects young women of Asian origin, typically less than 30 years of age. The disease manifests with fevers and unilateral lymphadenopathy that usually involves the cervical region in 70%-90% of affected patients, but axillary adenopathy has also been reported. The disease is also associated with systemic symptoms such as night sweats, rash, and weight loss. Laboratory abnormalities include leukopenia, neutropenia, elevated lactate dehydrogenase level, transaminitis, and an elevated erythrocyte sedimentation rate. The clinical and laboratory features frequently mimic lymphoma, infections, and autoimmune conditions such as SLE, scleroderma, autoimmune hepatitis, and thyroiditis. The definitive diagnosis of KFD is made via histopathology of the involved lymph node [6-8]. 

The pathogenesis of KFD remains ambiguous, but it has been reported that a viral or autoimmune etiology is most likely [8-10]. Viruses including Epstein-Barr virus, human herpesvirus 6, human herpesvirus 8, human immunodeficiency virus (HIV), parvovirus B19, paramyxoviruses, parainfluenza virus have been proposed as inciting agents. One study suggested a role for interferon-gamma and interleukin (IL-) 6 in the pathogenesis of KFD [11]. Several studies have described an association with autoimmune disorders, mainly SLE, scleroderma, and autoimmune hepatitis [12-14]. 

In our patient, KFD and SLE occurred concomitantly. It has been hypothesized that KFD may represent an initial phase of lupus, but the pathophysiological relationship remains unclear. KFD has been reported to precede, follow, or develop concomitantly with the diagnosis of SLE [8,12-13]. A study performed by Sopena et al. reported the association of Kikuchi-Fujimoto disease with SLE and found that SLE had been diagnosed before KFD in 18% of cases, simultaneously in 51%, and after KFD in 31% [8]. However, in a series of 244 people with KFD, only 32 were associated with SLE, suggesting that KFD may exist independently as well [14]. 

Given the considerable clinical overlap between the features of KFD and SLE, it may not be possible to differentiate between these entities at the initial presentation. Additionally, it is important to recognize that histologically, lupus lymphadenitis (LL) and KFD are difficult to distinguish [14,15]. The characteristic histopathological feature of KFD is histiocytic necrotizing lymphadenitis, characterized by karyorrhectic debris and histiocytic cellular infiltrates with central necrosis within the lymph node, often accompanied by invasion of the node capsule and inflammation of the perinodal tissue [6]. Similar findings are seen in LL, and some studies have suggested that the presence of neutrophils, hematoxylin bodies, and the Azzopardi phenomenon, wherein basophilic material is noted around blood vessels, are characteristics that favor LL. Yu et al. retrospectively studied the clinical and pathological features and found that C4d deposition, which reflected autoantibody-mediated complement activation, was noted in LL but not in KFD [15]. 

Our patient was initially diagnosed with KFD based on histopathology but subsequently met the diagnostic criteria for SLE. Differentiating KFD from its mimickers is imperative as clinical management varies. KFD is a self-limiting disease with the resolution of signs and symptoms in one to four months. Children with severe symptoms may benefit from glucocorticoids. However, patients with LL differ as they require aggressive management for disease control and prevention of end-organ damage. Therefore, with the lack of predictive markers to determine which cohort of patients with KFD are at risk of developing SLE, routine screening and close clinical follow-up for the development of SLE is crucial when histopathological differentiation cannot be performed. 

## Conclusions

We describe a female patient who presented with axillary lymphadenopathy and was initially diagnosed with KFD based on excisional lymph node biopsy, but later met the criteria for SLE. Lupus lymphadenitis and KFD share some common clinical and pathologic features. SLE may develop simultaneously with KFD and may precede or occur after KFD. Distinguishing between those two diseases can be challenging. When evaluating patients with KFD, it is crucial to recognize the relationship between SLE and KFD and carry out careful clinical surveillance to identify the potential coexistence of SLE and monitor for future development of SLE as the treatments differ considerably.

In our patient, a prompt biopsy of the affected lymph node, a high index of clinical suspicion, close outpatient follow-up, and monitoring allowed for the correct diagnosis was done. This case emphasizes the need to be aware that patients with SLE may present with lymphadenopathy and the need for clinicians to consider the association between Kikuchi disease and SLE to prevent misdiagnosis and allow for timely treatment to avoid complications.
